# Screening for organic acidurias and aminoacidopathies in high-risk Brazilian patients: Eleven-year experience of a reference center

**DOI:** 10.1590/1678-4685-GMB-2018-0105

**Published:** 2019-04-11

**Authors:** Moacir Wajner, Angela Sitta, Aline Kayser, Marion Deon, Ana C. Groehs, Daniella M. Coelho, Carmen R. Vargas

**Affiliations:** 1 Serviço de Genética Médica, Hospital de Clínicas de Porto Alegre, Porto Alegre, RS, Brazil; 2 Departmento de Bioquímica, Universidade Federal do Rio Grande do Sul, Porto Alegre, RS, Brazil; 3 Programas de Pós-Graduação em Ciências Biológicas, Bioquímica e em Ciências Farmacêuticas, Universidade Federal do Rio Grande do Sul, Porto Alegre, RS, Brazil

**Keywords:** Organic acidurias, aminoacidopathies, inborn errors of metabolism, selective screening

## Abstract

Organic acidurias and aminoacidopathies are groups of frequent inborn errors of metabolism (IEMs), which are caused by mutations in specific genes that lead to loss of protein/enzyme or transport function with important deleterious effects to cell metabolism. Since a considerable number of such disorders are potentially treatable when diagnosed at an early stage of life, diagnosis is crucial for the patients. In the present report, we describe symptomatic individuals referred to our service that were diagnosed with these disorders from 2006 to 2016. We used blood and urine samples from 21,800 patients suspected of aminoacidopathies or organic acidemias that were processed by the analytical techniques reverse phase high-performance liquid chromatography for amino acid quantification and gas chromatography coupled to mass spectrometry for organic acid detection. Analysis of dried blood spots by liquid chromatography-tandem mass spectrometry was used in some cases. We detected 258 cases of organic acidurias, and 117 patients with aminoacidopathies were diagnosed. Once diagnosis was performed, patients were promptly submitted to the available treatments with clear reduction of mortality and morbidity. The obtained data may help pediatricians and metabolic geneticists to become aware of these diseases and possibly expand newborn screening programs in the future.

## Introduction

Inborn errors of metabolism (IEM) are phenotypically and genetically heterogeneous disorders caused by defects in specific proteins, mostly enzymes, which compromise cellular metabolism. Although any given IEM is rare when taken individually, they are collectively numerous, causing significant morbidity and mortality (Mak *et al.*, 2013; Scaturro *et al.*, 2013). Prevalence of these disorders is probably underestimated, except were population-based neonate screening is conducted. More than 600 different IEMs have been already biochemically and molecularly identified. Disease state usually arises due to accumulation of toxic intermediate metabolites proximal to the metabolic block, lack of essential products, or accumulation of by-products (Scriver *et al.*, 2001). Clinical manifestations usually occur in early infancy and childhood, with a heterogeneous clinical spectrum.

Some IEMs, including amino acid, organic acid, and fatty acid oxidation defects, can be treated with promising outcomes. Affected babies are usually normal at birth, but symptoms may develop within hours or weeks pending on the severity of the disease. The symptoms are often non-specific, like poor feeding, seizures, and lethargy/coma that may progress to life-threatening complications if left untreated. Due to the serious clinical consequences for affected neonates, an early diagnosis allows pre-symptomatic treatment and is crucial in preventing irreversible mental retardation ranging from mild to severe, neurological damage, physical disability, and even death (Gramer *et al.*, 2014). Diagnosis of these disorders can be performed also for some prevalent and treatable IEM in the asymptomatic babies through newborn mass screening (NBS) that was established in the last three decades, or for most of these disorders through selective screening in symptomatic patients (Leonard, 2006; Marsdeen *et al.*, 2006).

Selective screening carried out in patients with clinical signs, alterations of routine laboratory tests, or family history indicating a metabolic disorder is an important diagnostic tool for the diagnosis of IEM in general. These tests usually measure small molecules in intermediary metabolism disorders whose concentrations are elevated in biological fluids due to a block at some point of the metabolic pathway. Other tests detect large complex molecules accumulating in lysosomal disorders, which have been incorporated more recently in battery of tests for selective screening (Bravo *et al.*, 2017). Improved diagnostic facilities for the detection of these disorders have increased the number of recognized IEM, as well as the discovery of new disorders. Early diagnosis is important not only for treatment but also for genetic counseling and prenatal diagnosis in future gestations. Late diagnosis and treatment of these metabolic disorders can cause permanent damage in essential tissues like the central nervous system (García-Gazorla *et al.*, 2009). The techniques used most commonly for the detection of small metabolites are gas chromatography-mass spectrometry (GC-MS) for organic acids determination, reverse phase high performance liquid chromatography (HPLC) and ion exchange chromatography-postcolumn derivatization for amino acids measurement (Joseph and Marsden, 1986), and liquid chromatography-tandem mass spectrometry (LC-MS/MS) for amino acid and acylcarnitine analysis (Rashed *et al.*, 1997). Basic metabolic tests comprise the assessment of urinary organic acids in urine, amino acids in plasma, urine and dried blood spots, as well as free carnitine and acylcarnitines in dried blood spots. The diagnosis is achieved by the identification of abnormal urinary organic acid profiling with elevated excretion of defined patterns of organic acids (organic acidurias), amino acids (aminocidopathies), and acylcarnitines (fatty acid oxidation defects) that are considered biomarkers for these classes of metabolic diseases.

Organic acidurias and aminoacidopathies are relatively frequent inherited disorders of intermediary metabolism resulting from deficiency of an enzyme or a transport protein involved in the catabolism of amino acids, carbohydrates, or lipids, leading to abnormal changes (usually increase) of physiological levels of organic acids and amino acids, respectively in the body and biological fluids (Van Vliet *et al.*, 2014). They are considered the most prevalent metabolic diseases in severely ill children and the most prevalent groups of IEM (Chalmers *et al.*, 1980). Many patients affected by these “intoxication” disorders have severe life-threatening symptoms with high morbidity and mortality. They typically suffer from either an acute life-threatening illness in early infancy or unexplained developmental delay with recurrent episodes of metabolic decompensation in later childhood. A wide variety of symptoms and laboratory findings occur, such as lethargy, coma, hypotonia, seizures, ataxia, vomiting, failure to thrive, developmental delay, liver disease, neutropenia, thrombocytopenia, hypoglycemia, hyperammonemia, etc. The possibility of providing an adequate treatment apparently justifies the detection of these diseases in patients without established diagnosis.

The true incidence of aminoacidopathies and organic acidurias in high-risk Brazilian patients is still poorly established. In this study, we present the prevalence of these diseases in patients suspected of IEM during the past eleven years (2006-2016) that were referred to our service, which is a regional and national reference center for the diagnosis of these diseases. Our objective was to find out the most prevalent organic acid and amino acid disorders in a high-risk Brazilian population and to promote the awareness of these disorders within the medical community as important and prevalent groups of genetic diseases.

## Subjects and Methods

### Patients

The methodology for the diagnosis of organic acidurias and aminoacidopathies was set up in 1994 at the Medical Genetics Service of the Hospital de Clínicas de Porto Alegre (HCPA), in the city of Porto Alegre, Southern Brazil. We aimed to provide an adequate treatment for a considerable number of these disorders, justifying therefore their detection in patients without established diagnosis. Plasma (1 mL) and fresh random urine specimens (2–10 mL) without preservative were collected and stored frozen within 2–3 h under –30 °C. Sometimes cerebrospinal fluid (CSF) was collected and frozen under the same conditions or dried whole blood spots in filter paper we used for amino acid detection. This study was approved by the Ethical Committee of Hospital de Clínicas de Porto Alegre. Patients who participated in this study or their parents and/or guardians signed informed consent forms for collection of the biological samples.

Samples were collected from symptomatic 21,800 children (52% boys and 48% girls) with suspected IEM or disorders of amino acid (plasma amino acid determination was requested in 48 % of samples) or organic acid metabolism (organic acid detection was requested in 52 % of urine samples), at the age varying from 24 h to 23 years from 17 Brazilian states (17/26 – 65.4%) from public and private hospitals and outpatient clinics. Children less than 3 years old were 66% of the sample, and less than 12 months old were 40%. Patients’ clinical information was obtained from a questionnaire filled by health professionals. Neurological features (81%), feeding refuse (21%), hepatomegaly (14%), dysmorphias (13%), vomiting (11%), and failure to thrive (5%) were the most frequent clinical manifestations, whereas hypoglycemia (15%), persistent or recurrent metabolic acidosis (13%), and lactic acidemia (6%) were the most common laboratory findings. Major neurological signs were unexplained psychomotor development/mental retardation (39%), persistent seizures (32%), abnormal muscular tone (22%), macrocephaly (3%), cerebral atrophy (2%), lethargy/coma (1%), and encephalopathic crises (1%).

### Amino acid analysis

The free amino acids in plasma, urine, or CSF were determined by reverse phase HPLC according to Joseph and Marsden (1986) with slight modifications (Wajner *et al.*, 2000). Plasma and CSF were precipitated by methanol, while sodium tetraphenylborate was added to urine and the supernatants were stored at –30 °C until analysis after centrifugation. A standard solution of amino acids was used for calibration (Sigma, Germany), prepared according to the manufacturer’s protocol. The analysis was performed using a Shimadzu HPLC with a reverse phase column (ODS 25 cm x 4.6 mm x 5 μm) and fluorescent detection after pre-column derivatization with orthophthaldialdehyde plus mercaptoethanol. The flow rate was adjusted to 1.4 mL/min in a gradient of the mobile phase (methanol and phosphate buffer: buffer A, 80% methanol; buffer B, 20% methanol). The duration of each analysis was 45 min. Amino acids were quantitatively determined by relating their chromatographic peak area to those obtained from a known standard mixture and to that of internal standard peak area (homocysteic acid). The results were reported as μmol/L.

### Organic acid analysis

Organic acids were determined in urine by GC/MS according to Sweetmann (2006), using hexadecane and heptadecanoic acid as internal standards. The organic acids were extracted twice with ethyl acetate and derived as bis(trimethyl-silyl) trifluoroacetamide plus trimethyl chlorosilane and identified as trimethyl-silyl compounds using a Agilent 7890A GC coupled to a Agilent 5975C MS detector with a capillary column DB-5MS (Agilent, length 30 m, internal diameter 0.25 mm, film 0.25 μ), with open-split injector and helium as carrier gas. GC/MS temperatures were as follows: column 90°C for 5 min until 280°C for 10 min (increment of 3°C/min), injector 250°C, transfer line 280°C, ion source 150°C, and mass analyzer 35°C. Finally, mass spectra was programmed from m/z 10 to 650 at the rate of 0.6 Hz.

### Acylcarnitine and amino acid analysis

Amino acids and acylcarnitines were analyzed in dried blood spot samples by liquid chromatography coupled to mass spectrometry in Tandem (LC/MS/MS) according to Rashed *et al.* (1997). Blood spot of 3 mm of diameter were mixed with 100 μL of a standard solution of deuterium-labeled acylcarnitines and amino acids. The acylcarnitines and the amino acids were analyzed as butyl esters using a 2695 Alliance (Waters) LC coupled to a Quattro Micro MS/MS (Perkin Elmer). Source and desolvation temperatures were 120 °C and 300 °C, respectively. Entrance energy, collision energy, and exit energy were 3V, 16eV, and 2V, respectively. Acylcarnitines and amino acids were monitored by MRM (multiple reaction monitoring), parent ion scan 85 (m/z) or neutral loss scan 102 (m/z). The concentrations were determined by the response of each analyte in relation to their respective labeled internal standard. The results were reported as μmol/L.

### Quality control

Our lab participates in the ERNDIM (European Research Network for Inherited Disorders of Metabolism) schemes for qualitative organic acid, quantitative amino acid, qualitative blood spot acylcarnitine as an external quality control program for accuracy evaluation.

## Results

Samples from 65% of referred patients came from six states of the southeast and south of Brazil that corresponds to the most prosperous Brazilian regions. We identified 258 cases of organic acid disorders and 117 patients with aminoacidopathies, making a positive diagnostic yield of about 1.72%. Tables 1 and 2 display the sex, average age at diagnosis, and major clinical and laboratory findings of amino acid and organic acid disorders, respectively. The average age at diagnosis was 3.8 years for aminoacidopathies and 4.6 years for organic acidurias. However, most diagnoses of amino acid metabolism disorders occurred before 1 year of age (64%), whereas 75% of the children were diagnosed before 3 years. Regarding organic acidurias, 49% were diagnosed before 1 year of age and 69 % before 3 years. Thus, many patients were diagnosed late with a high proportion of them being misdiagnosed initially as septicemia or meningitis. The delay of diagnosis had resulted in a significant morbidity such as mental retardation and physical handicaps among survivors in this cohort.

**Table 1:** Common features of patients with aminoacidopathies detected in high-risk Brazilians.

Age at diagnosis	Percentage
< 1 month	12
1 - 12 months	52
1 - 3 years	11
3 - 10 years	14
> 10 years	11
Gender	Percentage
Female	45
Male	55
Clinical features	Percentage
Neurological abnormalities	90
Feeding difficulties	30
Hepatomegaly	22
Vomiting	15
Dysmorphies	7
Failure to thrive	3
Neurological signs	Percentage
Psychomotor delay/mental retardation	61
Seizures	60
Hypotonia/hypertonia/dystonia	36
Cerebral atrophy	27
Macrocephaly	8
Laboratory findings	Percentage
Metabolic acidosis	41
Hypoglycemia	41
Lactic acidemia	14

**Table 2:** Common features of patients with organic acidurias detected in high-risk Brazilians.

Age at diagnosis	Percentage
< 1 month	5
1 - 12 months	44
1 - 3 years	20
3 - 10 years	17
> 10 years	14
Gender	Percentage
Female	47
Male	53
Clinical features	Percentage
Neurological	66
Vomiting	17
Feeding difficulties	15
Failure to thrive	6
Hepatomegaly	5
Dysmorphies	4
Neurological signs	Percentage
Psychomotor delay/mental retardation	30
Seizures	25
Hypotonia/hypertonia/dystonia	23
Macrocephaly	10
Laboratory findings	Percentage
Metabolic acidosis	32
Hypoglycemia	14

Table 3 shows the most prevalent aminoacidopathies and organic acidemias detected in our sample population. It should be emphasized that out of 18 children with urea cycle defects, 15 were diagnosed by LC/MS/MS, whereas only 3 by reverse phase HPLC. Table 4 compares the relative prevalence of organic acidurias in high-risk patients from different populations.

**Table 3:** Number and types of aminoacidopathies and organic acidurias detected in high-risk Brazilian patients from 2006 to 2016.

Amino acid disorders	Number	Percentage
Maple syrup urine disease	40	34
Phenylketonuria	24	21
Non-ketotic hyperglycinemia	17	14
Tyrosinemia	12	10
Homocystinúria	7	6
Citrullinemia	7	6
Ornithine transcarbamylase*deficiency*	4	3
Arginase *deficiency*	3	3
Carbamoyl phosphate synthetase *deficiency*	2	2
Argininosuccinic aciduria	1	1
Total	117	
Organic acid disorders		
Lactic acidemia	64	24.8
Glutaric aciduria type I	59	22.8
Methylmalonic aciduria	40	15.5
3-Hydroxy-3-methylglutaric aciduria	25	9.6
Isovaleric aciduria	12	4.6
Propionic aciduria	12	4.6
L-2-hydroxyglutaric aciduria	11	4.2
Alkaptonuria	10	3.8
Canavan disease	6	2.3
3-Methylglutaconic aciduria	4	1.5
D-2-hydroxyglutaric aciduria	4	1.5
3-Ketothiolase deficiency	4	1.5
Mevalonic aciduria	3	1.1
3-Methyl-Crotonyl-CoA Carboxylase deficiency	3	1.1
Glycerol Kynase deficiency	1	0.3
Total	258	

**Table 4:** Number and types of organic acidurias in different populations.

Disorders	Riyadh	Paris	San Diego	Freiburg	Asia	Singapore	India	Brazil
	(3 years)	(2 years)	(2 years)	(18 years)	(8 years)	(13 years)	(2 years)	(11 years)
Number of patients	76	77	41	90	111	24	45	258
Methylmalonic aciduria	31 (41%)	31 (40%)	20 (48%)	34 (37%)	74 (67%)	7 (29%)	15 (33%)	40 (15%)
Propionic aciduria	30 (39%)	21 (27%)	21 (51%)	33(36.6%)	23 (20%)	8 (33%)	16 (36%)	12 (5%)
Glutaric aciduria type I	10 (13%)	11 (14%)	ND	7 (8%)	6 (5%)	5 (21%)	ND	59 (23%)
Isovaleric aciduria	5 (7%)	14 (18%)	ND	16 (18%)	1 (0.9%)	ND	1 (2.2%)	12 (5%)
3-Hydroxy-3-methylgutaric aciduria	ND	ND	ND	ND	ND	ND	ND	25 (10%)

## Discussion

Rare disorders are neglected in most of developing countries due to other common infectious and other more prevalent diseases associated with malnutrition. However, rare disorders need to be investigated because a considerable number of patients in these countries suffers from IEM. Due to the huge burden of IEM, reference centers dedicated to selective screenings are important even for developing nations for the screening and diagnosis of the most prevalent IEMs such as organic acidurias and aminoacidopathies (Sass, 2011).

Analyses of organic acids and amino acids by sophisticated analytical methods are relatively expensive, especially considering the budget of developing countries. However, because of the relatively high prevalence of organic acidemias and aminoacidopathies among the known IEM (Chalmers *et al.*, 1980) and since many of these diseases can be successfully treated, we decided to implement the techniques for the diagnosis of these diseases at the Medical Genetics Service of the Hospital de Clínicas de Porto Alegre - a regional and national center of reference for studies on IEM. Early recognition of these metabolic diseases is also important when no treatment is currently available, for further family planning and possible prenatal diagnostics. In addition, awareness of these metabolic disorders by pediatricians, neurologists, and other physicians, as well as by the general public is also essential for the development of future therapeutic strategies. Finally, selective screening for IEM enables new disorders to be identified.

Therefore, in the present study, we investigated the relative prevalence of different organic acidurias and aminoacidopathies in clinically symptomatic Brazilian patients suspected of these pathologies. The major clinical and laboratory findings of the individuals referred to our service included unexplained neurologic signs, feeding difficulties, hepatomegaly, vomiting, failure to thrive, motor disabilities (hypotonia/hypertonia/dystonia), metabolic acidosis, hypoglycemia, and lactic acidosis. We incorporated in our laboratory 15 years ago an international quality control program for organic, amino acid and fatty acid determination that warrants a better reliability of our analysis, implying a good diagnostic approach for these disorders.

The use of a questionnaire containing a large spectrum of clinical and laboratory findings, as well as diet, medication, and age that was filled out by physicians requesting examinations for suspected patients helped us in the diagnosis of these diseases. Thus, these data is utterly important for correct assessment of chromatography findings on amino acids and especially organic acids, considering that there is a high variation on the level of these metabolites pending on age, clinical condition, and treatment at the time of sample collection. In our sample, no clinical and laboratory data were obtained from approximately 20% of the patients.

The average age of diagnosis (when patients were referred to our laboratory) was 3.8 years for aminoacidopathies and 4.6 years for organic acidurias, which could be attributed to lack of awareness of these genetic diseases by the physicians, lack of laboratories and expertise on the diagnosis of these disorders, high patient mortality for some disorders, or a combination of these factors. Nevertheless, the highest proportion of patients was diagnosed in the first year of life, whereas diagnosis of others disorders with lower mortality and less intense clinical manifestations such as L-2-hydroxyglutaric aciduria and glutaric acidemia type I had a late diagnosis.

All cases were confirmed in additional plasma, urine and CSF (patients with nonketotic hyperglycinemia) samples collected on at least two different occasions. For some disorders, we needed additional tests that were carried out in other laboratories especially in Europe and USA. Tandem MS was also performed in dried blood in some patients suspected of having urea cycle defects since HPLC does not discriminate certain amino acids and this enabled us to diagnose another fifteen cases of aminoacidopathies. Therefore, we strongly recommend this highly analytical method (LC/MS/MS) for the diagnosis of urea cycle defects.

Regarding the relative frequency of organic acidemias in our population, an interesting observation was the high number of glutaric aciduria type I (59 patients) and 3-hydroxy-3-methylglutaric aciduria (40 patients) detected in our study, with a similar prevalence to the other most prevalent organic acidurias, such as methylmalonic acidemia (due to mutase deficiency or various cobalamin deficiencies including CblC and Cbl A). (Borden, 1984; Lehnert and Niederhoff, 1984; Saudubray *et al.*, 1989; [Bibr B19] Rashed *et al.*, 1994; Hori *et al.*, 2005; Tan *et al.*, 2006; Narayanan *et al.*, 2011; Al Riyami *et al.*, 2012; Jiang *et al.*, 2015; Lampret *et al.*, 2015). In contrast, the number of propionic acidurias in our population was below the expected and this may have occurred because of the severity and high mortality of the affected patients so that some of them were not referred to our service. Regarding 3-hydroxy-3-methylglutaric aciduria, we have previously reported that some mutations observed in our population were similar to the Portuguese population; by looking at the surnames, all but one patient had Portuguese origin (Vargas *et al.*, 2008).

Of the 64 cases of primary lactic acidemias diagnosed (high lactic aciduria and lactic acidemia) detected on three consecutive occasions, only a few of them had the biochemical and/or the molecular defect defined (including deficiencies of glucose-6-phosphatase deficiency, fructose-1-6-biphosphatase, pyruvate dehydrogenase, and pyruvate carboxylase), indicating the difficulty of establishing the biochemical cause in this group of disorders. Furthermore, approximately half of these children died suddenly at the time of diagnosis or a few weeks later, reflecting the severity of these mitochondrial disorders associated with the lack of effective therapy. On the other hand, since most patients affected by organic acidurias lived in other states in Brazil, we did not have their follow up in order to determine precisely the rate of mortality for these diseases. Nevertheless, all diagnosed patients were immediately submitted to the available treatment mainly based on restricted diets and supplementation of vitamins, amino acid mixture, glycine and/or L-carnitine, and liver transplantation for some those affected by aminoacidopathies as maple syrup urine disease and tyrosinemia type I after all treatment efforts failed. Furthermore, the clinical symptomatology of our patients with organic acidurias was often severe probably because of the late diagnosis of which outcomes are much worse. This was particularly observed in glutaric aciduria type I, in which striatum degeneration affected the vast majority of our patients and represents an irreversible and crucial step towards spasticity and other marked debilitating muscular symptoms.

Another aspect that seems worth mentioning is that specimens for selective organic acid screening need to be collected preferentially during a crisis of metabolic decompensation usually caused by catabolic events triggered by intercurrent infections, changes in nutrition, or prolonged fasting (*e.g.*, the first urine). Sampling after recovery, even if it is only partial, may result in decreased sensitivity and may not reveal diagnostic abnormalities detectable before recovery.

Regarding aminoacidopathies, we diagnosed a relatively high number of maple syrup urine disease (40), nonketotic hyperglycinemia (17), and tyrosinemia type I (12), probably because of the severity of the clinical findings of these disorders, which raises the awareness of pediatricians and neurologists. Of the urea cycle defects cases, only three were diagnosed by HPLC, and 15 additional patients with these disorders were diagnosed by LC/MS/MS. Thus, it is emphasized that although important, HPLC does not allow complete separation and/or identification of all amino acids that can be otherwise identified by amino acid analysis on LC/MS/MS, including arginine and citruline. Interestingly, 20 cases of PKU were referred to our laboratory for confirmation because of abnormal tests (high phenylalanine levels) in the first sample at private laboratories also dedicated to the NBS program, whereas we diagnosed four cases of PKU in mentally retarded children that probably escaped the NBS detection of this disorder, whose nationwide coverage is about 86% in Brazilians babies.

Finally, we emphasize that some patients with organic acid disorders and aminoacidopathies were inadvertently confounded with septicemia, which also presents severe symptoms such as acidosis and acute encephalopathy; these late diagnoses often lead to death or permanent sequelae. Our results indicate the importance of diagnosing organic acidemias and aminoacidopathies *in loco* and as early as possible even in a developing country and particularly in severely ill patients because of the high prevalence of these diseases in these high-risk groups of patients, and particularly because of the availability of therapy for many of them. Treatment monitoring of affected individuals by serial analysis of urine and blood metabolites is also important and justifies the setup of such facilities despite extra costs. Our study shows that amino acids and organic acids disorders may warrant the development of a nationwide screening program for the diagnosis of these disorders because of their high incidence in the neonatal period in Brazil. Thus, we propose that the NBS program should be expanded in Brazil to include these disorders, besides PKU, in neonates in order to avoid the high morbidity and mortality, ensuring easy and equal access to this preventive public health initiative and to increase the awareness and knowledge among the general public and especially, health professionals. Further studies are needed to investigate the long-term outcome and costs associated with the diagnosis and treatment of these disorders.
